# Non-coding RNAs in cancer: multi-omics insights, liquid biopsy advances, drug resistance mechanisms, and the road to clinical translation

**DOI:** 10.3389/fonc.2026.1924660

**Published:** 2026-08-20

**Authors:** Şeyma Tutar, Vida Rezayifar, Hamida Abakar Mohamed Elhaj, Merve Tutar, Cennet Ataman, Lütfi Tutar

**Affiliations:** 1 Division of Biochemistry, Faculty of Medicine, Recep Tayyip Erdogan University, Rize, Türkiye; 2 Division of Clinical Biochemistry, Faculty of Medicine, Recep Tayyip Erdoğan University, Rize, Türkiye; 3 Department of Biology, Faculty of Mathematics and Natural Sciences, University of Cologne, Cologne, Germany; 4 Molecular Biology Laboratory (Food Analysis Department), Lifeprint GmbH, Illertissen, Germany; 5 Departmant of Molecular Biology and Genetics, Faculty of Science and Letters, Kırşehir Ahi Evran University, Kırşehir, Türkiye

**Keywords:** antisense oligonucleotides, clinical trials, drug resistance, immune modulation, liquid biopsy, non-coding RNAs, personalized medicine, therapeutic target

## Abstract

For most of the twentieth century, the transcriptional output of the human genome was thought to be biologically inert—a characterization that has been proven wrong in almost every important respect. Non-coding RNAs (ncRNAs) such as microRNAs (miRNAs), long non-coding RNAs (lncRNAs), circular RNAs (circRNAs), small nucleolar RNAs (snoRNAs) and PIWI-interacting RNAs (piRNAs) are now thought of as vital regulators of gene expression in all the stages of cancer pathogenesis, including the initial epigenetic changes, metastatic spread and the development of therapeutic resistance. This review highlights four areas where the clinical potential of ncRNAs is most promising: reconstruction of ncRNA regulatory networks by multi-omics integration; circulating ncRNAs as minimally invasive cancer biomarkers; causal roles of ncRNAs in drug resistance through epithelial-mesenchymal plasticity, metabolic reprogramming, and stromal communication; and translation of ncRNA targeting strategies to clinical trials. We will need to invest equally in mechanistic rigor and translational infrastructure to move forward.

## 1 Introduction

The realization that >80% of the human genome is actively transcribed, although <2% of transcripts encode proteins, has initiated a paradigm shift in molecular biology (1). What had been thought of as genomic background proved to be a layer of regulation of extraordinary complexity, replete with ncRNAs whose functional diversity exceeds, and often surpasses, the diversity of the protein-coding genes they were once thought to flank without purpose (2). Nowhere has this reorientation had more practical consequence than in cancer biology. Tumorigenesis results from the additive failure of gene regulation, and ncRNAs are involved in virtually all clinically relevant regulatory failures. They silence tumor suppressors by promoter methylation and chromatin remodelling. They sequester inhibitory miRNAs to sustain oncogenic signaling. They are involved in remodeling of the tumor immune microenvironment. And they provide the transcriptional plasticity to cancer cells that is required for them to survive chemotherapy, targeted therapy and immunotherapy (2). This ubiquity makes ncRNAs attractive targets for therapeutics but also complicates interpretation of any single mechanistic study.

Two technological advances have taken ncRNA-based medicines one step closer to clinical reality. First, tumor-derived exosomes package ncRNAs in a form that survives circulation in blood, urine, and cerebrospinal fluid, thus enabling non-invasive tumor sampling that circumvents the spatial and temporal limitations of tissue biopsy. Second, the regulatory approval of patisiran demonstrated that RNA-based drugs can be formulated for systemic delivery in humans, a pharmacological precedent that is now being extended to oncology (3–8).

## 2 ncRNA classification, biogenesis, and cancer-relevant functions

### 2.1 MicroRNAs

miRNAs are 22 nucleotides long. Mature miRNAs are incorporated into the RNA-induced silencing complex (RISC), which is guided to complementary sequences in the 3’ untranslated regions of mRNAs leading to translational repression or transcript degradation (2). Their biogenesis is well described. Primary transcripts are processed in the nucleus by Drosha and DGCR8 (DiGeorge syndrome critical region 8), exported as pre-miRNAs, cleaved by DICER and loaded strand-selectively into AGO2 (Argonaute 2). One miRNA can regulate hundreds of different mRNAs, and roughly 60% of protein-coding genes contain predicted miRNA binding sites, meaning that any change in miRNA expression can have a disproportionate effect on cellular phenotype (2).

miR-21 is overexpressed in colorectal, lung, breast, and hematologic cancers and promotes cell survival and resistance to 5-fluorouracil by silencing PTEN and PDCD4. miR-34a has the reverse functional role – a direct transcriptional target of p53 that elicits G1 arrest and apoptosis through repression of CDK4, CDK6, BCL2 and SIRT1. One of the most common epigenetic lesions in human tumors is the hypermethylation of the miR-34a promoter, which reflects the selective pressure cancer exerts to silence this suppressor. miRNAs are attractive biomarker candidates because of their stability in biofluids, which is due to their binding to Argonaute and encapsulation in exosomes (9–13).

### 2.2 Long non-coding RNAs

lncRNAs are >200 nucleotides long and represent a highly heterogeneous functional class. They act through multiple mechanisms, including guiding PRC2 or LSD1 to specific genomic loci and serving as scaffolds for transcription factor complexes at gene promoters. They also function as competing endogenous RNAs (ceRNAs) that sequester miRNAs and play a role in regulating alternative splicing (5). HOTAIR recruits PRC2 to mediate H3K27me3-mediated silencing of metastasis suppressors and independently prognosticates relapse in breast cancer (14–17).

Two lncRNAs from original articles in this Research Topic exemplify the mechanistically specific lncRNA cancer biology that has emerged. Juskiene et al. showed that the MALAT1 rs619586 G allele is associated with susceptibility to pituitary adenoma and more invasive, recurrence-prone disease (18). In another cancer setting, LINC02178 was shown to inhibit ferroptosis in HPV16-positive cervical cancer cells by up-regulation of PLIN2 and inhibition of lipid peroxidation, and silencing LINC02178 leads to ROS accumulation, glutathione depletion and a sharp decrease in the ferroptosis threshold, with direct pharmacological implications for combination strategies with ferroptosis inducers (19).

### 2.3 Circular RNAs

circRNAs are generated by back-splicing, producing covalently closed loops that resist exonucleolytic degradation. As a result, they accumulate to higher steady-state levels than linear transcripts. The structural stability makes them attractive as liquid biopsy analytes. The most studied functional role of circRNAs is miRNA sponging. However, RNA-binding protein sequestration, translation of stress-induced peptides, and parental gene regulation have also been documented (3). Quantitative evidence for endogenous ceRNA activity remains more limited than the literature would suggest; this point is discussed in the Discussion.

### 2.4 snoRNAs and piRNAs

snoRNAs guide site-specific modifications of ribosomal RNA: 2’-O-methylation by C/D box snoRNAs and pseudouridylation by H/ACA box snoRNAs and have long been considered as constitutive housekeeping factors. That view has been seriously altered. The expression of numerous snoRNAs is dysregulated in lung, colorectal, and hematological malignancies. A systematic review in 2025 documented roles of snoRNAs, tsRNAs and snRNAs in modulating PD-1/PD-L1, CTLA-4, Tim-3 and LAG-3 expression, T cell polarization and macrophage phenotype (8). A snoRNA-based tumor immune infiltration score in colorectal cancer was associated with poor prognosis and poor ICI response even in MSI-H patients, which is a clinically relevant finding that may add information to improve patient selection beyond current MSI/TMB criteria (20–23).

piRNAs, generated via PIWI-associated ping-pong amplification, have long been considered absent from significant somatic cancer regulation. Since then, dysregulated piRNA-PIWI complexes have been connected to aberrant epigenetic reprogramming, maintenance of cancer stemness and EMT-driven drug resistance, all reported in several tumor types as actionable targets in resistant disease (24–28). These molecules were absent from early reviews of ncRNA cancers, as the tools to detect them did not exist, but that gap is narrowing (**Table 1**).

**Table 1:** Major classes of non-coding RNAs, their structural features, and core mechanisms of action in cancer.

ncRNA class	Size/structure	Core mechanism(s)	Cancer-relevant example
miRNAs	~22 nucleotides	Loaded into RISC, guides complex to 3’UTR sequences causing translational repression or degradation	miR-21 (oncogenic, silences PTEN/PDCD4); miR-34a (tumor suppressor, p53 target)
lncRNAs	>200 nucleotides	Guides PRC2/LSD1 to genomic loci, scaffolds transcription factor complexes, acts as ceRNA, regulates splicing	HOTAIR (recruits PRC2, silences metastasis suppressors); MALAT1 (pituitary adenoma susceptibility)
circRNAs	Covalently closed loops (back-spliced)	miRNA sponging, RNA-binding protein sequestration, stress peptide translation, parental gene regulation	LINC02178-related ferroptosis regulation in cervical cancer
snoRNAs	Small nucleolar RNAs	Guide rRNA 2’-O-methylation (C/D box) and pseudouridylation (H/ACA box); immune modulation (PD-1/PD-L1, CTLA-4)	TIIsno immune score in colorectal cancer
piRNAs	PIWI-interacting, ping-pong amplification	Epigenetic reprogramming, cancer stemness maintenance, EMT-driven resistance	piRNA-PIWI complexes in resistant tumor types

## 3 Multi-omics integration: building mechanistic ncRNA networks

### 3.1 Beyond single-platform transcriptomics

Single-platform RNA-seq can show changes in expression but not why they happened. ncRNAs act at the intersection of chromatin state, transcription factor occupancy, RNA processing efficiency and protein-RNA binding - all of which require a separate layer of analysis to resolve. Integrating RNA-seq with ATAC-seq, histone modification ChIP-seq, and CLIP-seq enables reconstruction of cell type-specific ncRNA regulatory networks that cannot be inferred from transcriptomic data alone that are not visible to expression-only approaches (2). An integrative analysis of TCGA transcriptomic data and ENCODE chromatin datasets identified lncRNA-transcription factor co-regulatory circuits controlling invasion and therapy resistance in breast, lung, and colorectal cancers (2, 29–32).

Add to that the epitranscriptome, another level of complexity. N6-methyladenosine (m6A) modifications, which are deposited by METTL3/METTL14 and read by YTHDF proteins, change the stability, subcellular localization, and protein-binding capacity of lncRNAs and circRNAs in ways that standard RNA-seq does not capture (2, 5). In glioblastoma and AML, m6A-hypermethylated ncRNAs maintain oncogenic programs that are compromised by pharmacologic inhibition of METTL3 activity, a finding now driving early clinical testing of METTL3 inhibitors (33–36).

### 3.2 Single-cell resolution and the ceRNA evidence problem

Single-cell multiome sequencing, combining scRNA-seq and scATAC-seq on the same cell, profiles ncRNA expression to specific chromatin accessibility states in individual tumor cells, identifying regulatory programs in therapy-resistant subclones that bulk analyses cannot. Spatial transcriptomics anchors these maps in tissue architecture and reveals significant differences in ncRNA profiles in the hypoxic core, invasive margin and cancer-immune interface.

The volume of ceRNA literature far exceeds the quantitative evidence supporting the hypothesis in most cellular contexts, and a measured appraisal of the hypothesis is warranted. Most endogenous lncRNA molecules are present at concentrations that would not be expected to compete significantly with mRNAs for shared miRNA target sites, as determined by thermodynamic modeling and single cell copy number analyses. ceRNA activity is real under defined conditions, particularly when competing RNA levels are elevated through amplification or overexpression, but should not be inferred from sequence-based network predictions (3, 37–39). Future studies should adopt more rigorous quantitative and functional criteria before pursuing a therapeutic logic based on ceRNA at clinical scale.

## 4 Liquid biopsy: circulating ncRNAs as tumor biomarkers

### 4.1 Technical landscape and standardization gaps

It is technically not difficult to detect ncRNAs in blood, it is technically difficult to detect them reproducibly across laboratories. The quantitative variability introduced by pre-analytical variables such as collection tube anticoagulant, centrifugation protocol, exosome isolation method, RNA extraction kit, and cDNA synthesis conditions can mask biologically real between-group differences (3). To date, no endogenous reference ncRNA has demonstrated stability across different cancer types, disease stages, and common comorbidities for clinical-grade circulating assays. Unless international pre-analytical consensus standards are established, multi-site biomarker studies will continue to produce non-reproducible results. Detection platforms include quantitative RT-PCR, digital droplet PCR, and attomolar-sensitive CRISPR-Cas12/13-based assays that could allow for eventual point-of-care deployment (3, 40–43).

### 4.2 Diagnostic and pharmacodynamic evidence

Tumor-derived exosomes concentrate ncRNAs from particular cell populations and protect them from circulating RNases. Serum exosomal lncRNA FOXD2-AS1 had AUC 0.758 for early colorectal cancer, which was better than carcinoembryonic antigen and CA19–9 at that stage of disease (44, 45). A machine learning signature comprised of four lncRNAs from gastric cancer exosome RNA-seq data achieved AUC 0.959, 0.942 and 0.949 in training, test and external validation cohorts, respectively, including patients with completely normal conventional tumor marker results (46). Multi-analyte circRNA panels for non-small cell lung cancer demonstrated greater classification accuracy than any individual circRNA marker tested alone (3).

In the PROSPECT-R trial (NCT03010722), serial measurement of circulating miR-652-3p tracked treatment response and predicted emerging regorafenib resistance in metastatic colorectal cancer (47). This pharmacodynamic application—quantifying an active tumor regulatory process in real time—may have more immediate clinical utility than early detection, which requires much larger validation studies before changing clinical practice. This is because pharmacodynamic biomarkers typically require smaller validation cohorts and can demonstrate clinical utility earlier than screening biomarkers, which must be validated across large, heterogeneous populations before detecting disease reliably.

## 5 ncRNAs and the tumor immune microenvironment

### 5.1 ncRNA-driven immune evasion

The TME is actively programmed by ncRNA-loaded extracellular vesicles that are continuously circulating between cancer cells, TAMs, CAFs, T and B lymphocytes, and NK cells (48–50). NEAT1 promotes NF-κB activation and M2-like macrophage polarization. PD-L1 is up-regulated by PVT1 in several tumor types. Tumor-secreted miR-934 and miR-183 reprogram macrophage phenotype to immunosuppressive, establishing a feed-forward loop in which the tumor actively maintains the immune environment that protects it (51–53). Slack and Chinnaiyan reported the wide range of lncRNAs that can change immune cell infiltration patterns in different cancers (53).

More than previously suspected, snoRNAs, tsRNAs, and snRNAs are more directly involved in immune reprogramming. Liu et al. reported their functions in regulating PD-1, PD-L1, CTLA-4, Tim-3 and LAG-3 expression, T cell polarization and tumor antigen presentation. The snoRNA-based TIIsno score predicted poor ICI response even in MSI-H colorectal cancer patients—challenging the assumption that mismatch repair deficiency alone identifies ICI responders and raising the possibility that snoRNA immune profiling could meaningfully refine current patient selection criteria (23).

### 5.2 CAF-mediated stromal resistance

CAF-derived exosomal ncRNAs are increasingly recognized as important contributors of acquired treatment resistance. These cells can stimulate Wnt/β-catenin, STAT3 and PI3K/AKT survival pathways in neighboring cancer cells, induce MDR1 and ABCG2 drug efflux transporters, inhibit ferroptotic susceptibility and augment PD-L1 expression to reduce T cell infiltration (54). A single exosomal ncRNA from CAFs can simultaneously influence chemotherapy, targeted therapy and immunotherapy responses. It is this breadth that makes this communication axis clinically difficult and therapeutically attractive as a multi-resistance target.

## 6 ncRNA-mediated drug resistance

### 6.1 Three axes of resistance plasticity

Chen et al. grouped the ncRNA contributions to drug resistance into three related plasticity axes (24). Identity plasticity, the acquisition of mesenchymal and stem-like phenotypes, occurs through ncRNA-driven EMT — lncRNAs such as MALAT1 and HOTAIR activate ZEB1/2 and Snail, while miR-200 family members, which normally suppress these factors, are simultaneously downregulated (18, 24). State plasticity is characterized by metabolic and transcriptional reprogramming: ncRNAs reprogram glycolysis, glutamine utilization and mitochondrial function in a way that reduces drug uptake and diminishes apoptotic sensitivity. Communication plasticity is mediated by exosomal ncRNA transfer between cell populations, spreading resistance programs horizontally through the tumor ecosystem (24).

Li et al. demonstrated that miR-2110 suppresses ERK–ELK1 transcriptional activity in AML, resulting in G1 arrest and cooperation with cytarabine in drug-resistant cell lines and primary patient samples (55). A miR-2110 mimic can re-sensitize refractory patients, as cytarabine is already standard of care, no new drug target needed. Chen et al. demonstrated that LINC02178 supports the viability of cervical cancer cells, at least in part, through inhibiting the execution of ferroptosis. LINC02178 depletion leads to the surge of ROS, malondialdehyde accumulation, increase of ferrous iron and depletion of glutathione that act in concert to push cells across the ferroptotic threshold (19). Combining LINC02178 inhibition with RSL3, erastin, or sorafenib is a mechanistically grounded combination hypothesis that warrants formal preclinical development.

### 6.2 Pharmacological reversal strategies

Restoring tumor suppressor miRNA function through LNP-delivered synthetic mimics shows preclinical synergy with cisplatin, taxanes, and EGFR inhibitors, primarily by re-engaging apoptotic pathways and blocking EMT transcription factors. GapmeR ASOs targeting oncogenic lncRNAs reduce PD-L1 expression and can partially restore tumor immunogenicity in preclinical ICI combination models (4, 14). Naturally occurring compounds — curcumin upregulating miR-206 to inhibit JAK/STAT3, β-elemene suppressing miR-1323 to prevent EGFR-driven EMT, berberine modulating lncRNA circuits to reduce stemness — demonstrate proof-of-concept for ncRNA-mediated resistance reversal, though achieving therapeutic tissue concentrations in patients remains a genuine pharmacokinetic challenge (24).

## 7 ncRNA-targeted therapeutics: clinical trial evidence

### 7.1 The pharmacological toolkit

ASOs containing a phosphorothioate backbone and 2′-modified sugars (LNA or MOE) silence ncRNA targets by steric blocking or RNase H-mediated cleavage of RNA: DNA duplexes (56). LNA-modified antimiRs represent the most clinically advanced class of antimiRs, with a first-in-human trial of an LNA antimiR against miR-155 showing target engagement and early anti-tumor activity in refractory solid tumors (4). siRNAs encapsulated in LNPs downregulate protein-coding effectors downstream of oncogenic ncRNA circuits. miRNA mimics replace function of silenced tumor suppressor miRNAs. saRNAs enhance the transcriptional expression of tumor suppressor genes by targeting promoter-associated ncRNAs, an approach that has now progressed to clinical testing in HCC (4, 56).

### 7.2 Delivery: still the central problem

LNPs are the most validated clinical delivery platform for RNA drugs, but their pharmacokinetics are biased toward liver accumulation (56). This has essentially restricted the most sophisticated ncRNA therapeutics to hepatic targets. Active surface targeting with folate, aptamer, or antibody fragments improved tumor selectivity in animal models, but clinical-grade production of targeted formulations at scale remains unsolved. SNA technology has also started to overcome another challenge, the blood-brain barrier (57). The bottom line is that delivery to extrahepatic solid tumors is the main reason for fewer actionable results in ncRNA oncology trials than predicted by preclinical data.

### 7.3 Key clinical trial findings

Advanced solid tumors with liver involvement were treated with an LNP formulation co-delivering siRNAs against VEGF-A and KSP. Biopsies confirmed siRNA-mediated mRNA cleavage at both hepatic and extrahepatic tumor sites, the first evidence in humans that systemic RNAi reaches tumor tissue. A confirmed complete response was observed in one heavily pretreated patient (58). We found an unexpected and clinically important extra-hepatic activity.

Tolerability of liposomal siRNA targeting PKN3 was demonstrated in Phase I monotherapy (59). The subsequent phase 1b/IIa combination study with gemcitabine in metastatic pancreatic cancer demonstrated median PFS of 5.33 compared to 1.81 months with twice-weekly versus once-weekly dosing (p = 0.025 in the metastatic subgroup), with disease control in seven of 12 patients in the twice-weekly arm (60). Those numbers are a real clinical signal in pancreatic adenocarcinoma.

SNA nanoconjugates carrying Bcl2L12-targeting siRNA were administered intravenously to patients with recurrent glioblastoma prior to tumor resection. Transmission electron microscopy confirmed blood–brain barrier penetration and SNA accumulation within the tumor. Bcl2L12 protein was significantly lower in resected tumor tissue. No grade 4 or 5 adverse events were reported (57). This Phase 0 study yielded the first clinical evidence of SNA technology as a breach to the BBB.

Liposomal miR-34a mimic — designed to restore the most commonly silenced tumor suppressor miRNA — produced three partial responses in advanced HCC and solid tumors, and showed dose-dependent target gene signature changes in peripheral blood cells (61). Early termination was motivated by five serious immune-mediated adverse events (four deaths) attributed to complement activation by the lipid formulation. That result directly influenced subsequent RNA therapeutic trial design: complement safety monitoring and modified LNP chemistry have been standard in the field ever since.

saRNA technology transcriptionally restored C/EBPα expression in advanced HCCs. Target engagement was confirmed in peripheral blood mononuclear cells and the safety profile was acceptable – establishing that RNA-driven gene activation, not only silencing, is attainable in cancer patients (4).

TargomiR minicells targeting EGFR carrying miR-16 mimic reached patients with malignant pleural mesothelioma. In 22 evaluable patients there was one partial response and 15 stable disease. Biopsies confirmed the accumulation of miRNA at the tumor site. No dose-limiting toxicities related to the miRNA payload were observed (62). TargomiR demonstrated that engagement of ncRNA targets is feasible in human tumors using non-lipid delivery platforms (**Table 2**).

**Table 2:** Non-coding RNAs implicated in drug resistance: mechanisms, therapeutic strategies, and clinical trial evidence.

ncRNA/Target	Resistance mechanism	Therapeutic strategy	Clinical trial evidence
MALAT1, HOTAIR	EMT via ZEB1/2, Snail activation (identity plasticity)	GapmeR ASOs targeting oncogenic lncRNAs	Preclinical/early-stage combination with ICIs
miR-200 family (loss)	Downregulation permits EMT progression	miRNA mimic restoration	Preclinical synergy with cisplatin, taxanes, EGFR inhibitors
miR-2110	Suppresses ERK-ELK1, G1 arrest reversal in AML	miR-2110 mimic + cytarabine	Demonstrated re-sensitization in refractory patient samples
CAF-derived exosomal ncRNAs	Wnt/β-catenin, STAT3, PI3K/AKT activation; MDR1/ABCG2 induction	Targeting stromal communication axis	Preclinical stage
miR-155 (LNA antimiR)	Oncogenic miRNA overactivity	LNA-modified antimiR	First-in-human trial showing target engagement in refractory solid tumors
siRNA *vs* VEGF-A/KSP (ALN-VSP02)	N/A (delivery proof-of-concept)	LNP-delivered siRNA	Confirmed complete response in one heavily pretreated endometrial cancer patient
PKN3 siRNA (liposomal)	Pancreatic cancer progression	Liposomal siRNA + gemcitabine	Phase 1b/IIa: median PFS 5.33 *vs* 1.81 months (twice- *vs* once-weekly dosing)
Bcl2L12 siRNA (SNA nanoconjugate)	Glioblastoma recurrence	SNA nanoconjugate, BBB-penetrant	Phase 0: confirmed BBB penetration, reduced Bcl2L12 protein
miR-34a mimic (MRX34)	Loss of tumor suppressor function	Liposomal miR-34a mimic	3 partial responses in HCC/solid tumors; halted due to immune-mediated adverse events
CEBPA (saRNA, MTL-CEBPA)	Loss of differentiation signal	saRNA transcriptional activation	Confirmed target engagement in advanced HCC, acceptable safety
miR-16 mimic (TargomiR)	EGFR-driven resistance in mesothelioma	EGFR-targeted minicells carrying miR-16 mimic	1 partial response, 15 stable disease in 22 evaluable patients

## 8 Artificial intelligence and emerging technologies

The ncRNA interactome – which contains hundreds of thousands of lncRNAs, over 100,000 annotated circRNAs and millions of predicted binding interactions – is too large for hypothesis-driven experimental programs capable of systematic interrogation. Graph neural networks trained on multi-omics cancer datasets identify regulatory hub ncRNAs and predict context-specific interaction activity with an accuracy that cannot be matched by conventional network analysis (23). Deep learning models predict ncRNA secondary structure, CLIP-seq protein partners and disease associations from sequence features, allowing for rational prioritization of ncRNA drug targets prior to committing resources to experimental validation.

Federated learning provides a practical solution to the persistent problem of small, institution-specific datasets, especially for rare tumor types (23), by allowing biomarker models to be trained across institutional cohorts without centralizing patient data. CRISPR-Cas13 enables programmable *in vivo* ncRNA knockdown with preclinical antitumor efficacy in lung cancer and glioblastoma models. Coupled with genome-scale CRISPRi/a screens for systematic ncRNA functional annotation, these tools are paving the way for a more evidence-based target discovery pipeline than the field has ever known.

## 9 Discussion

### 9.1 Where the field still struggles

Pre-analytical standardization is not a minor technical concern, but a scientific validity concern. The measurement of the same biomarker in circulating ncRNA is often contradictory across sites, much of this variability is due to undisclosed differences in sample handling. No endogenous reference ncRNA is validated for stability across cancer types, disease stages, and common comorbidities for clinical-grade assays. Without international agreement on standards for collection and processing prior to the initiation of Phase III biomarker trials, the liquid biopsy literature will continue to grow with promising but non-reproducible findings.

The MRX34 trial is the most important safety lesson in ncRNA therapeutics to date. Improvements in LNP formulations and chemical modifications since 2016 have largely eliminated innate immunostimulatory properties, and complement screening is now standard, but the risk of serious immune activation from systemic RNA delivery persists (4, 63). This represents a true safety constraint that should inform trial design as more potent formulations are developed for larger patient populations.

We need to directly confront the stoichiometric problem of ceRNA biology. Many published lncRNA and circRNA cancer studies rely on ceRNA logic from computational network analysis without addressing whether the implicated sponge molecules are expressed at levels sufficient to compete for relevant miRNAs in the cell type of interest (4). While ceRNA activity is real in specific defined conditions, taking it as the default mechanism without quantitative validation represents a reproducibility problem that the field should proactively address.

### 9.2 Near-term opportunities

The most tractable near-term translational target is circulating ncRNA pharmacodynamic monitoring. Analytical and clinical validation studies, not full drug development pipelines, are needed to validate ncRNA treatment response signatures, and the PROSPECT-R precedent demonstrates this can be accomplished within existing trial infrastructure (4). This approach is realistic to be applied to monitoring of targeted therapy resistance and prediction of ICI response within five years.

Next-generation LNP formulations with improved endosomal escape, tumor-specific surface ligands and reduced innate immunogenicity are moving into preclinical development and early clinical testing (56). The idea of engineered exosome platforms harnessing natural tumor-homing capacity is tantalizing; the manufacturing challenge is real, but not fundamentally different from what gene therapy faced a generation ago. circRNA-based ncRNA neoantigen vaccines integrate the structural stability of circRNAs with the delivery of peptide antigens in a potentially more effective design that could elicit more durable antitumor immune responses than current mRNA vaccine platforms.

snoRNAs and piRNAs are an under-invested research area with real clinical potential. The TIIsno findings in colorectal cancer (23) and the piRNA-PIWI axis in EMT-driven resistance (24)suggest that these classes hook into cancer regulatory programs at nodes not targeted by existing miRNA- or lncRNA-focused strategies. Targeted investment in these underexplored groups likely will uncover new starting points for diagnostics and therapeutics.

## 10 Conclusion

The importance of ncRNAs in cancer biology is well deserved. The evidence presented here, including multi-omics network reconstruction and liquid biopsy diagnostics, TME reprogramming, drug resistance mechanisms and clinical trial results, shows that these molecules are core components of the oncogenic machinery, individually targetable and collectively necessary to tumor survival and adaptation.

The clinical translation record is mixed but real. ALN-VSP02 proved that systemic RNAi can work in cancer patients (58). Blood–brain barrier penetration of NU-0129 (57). MRX34 showed tumor-site pharmacodynamic engagement and identified a critical safety liability (61). Clinical feasibility of saRNA gene activation was demonstrated with MTL-CEBPA (4). Every one of these results, including the failures, changed the design of the next generation of trials, and that is real scientific progress.

The three original research articles in this Research Topic -- Li et al. on miR-2110 and AML cytarabine sensitivity (55), Chen et al. on LINC02178 and cervical cancer ferroptosis (19), and Juskiene et al. on MALAT1 and pituitary adenoma susceptibility (18) -- exemplify what mechanistically specific, clinically contextualized ncRNA research looks like in practice. They discover molecular weaknesses in certain kinds of tumors whose therapeutic implications are not abstract or far-off. It is this combination of mechanistic depth and translational relevance that drives ncRNA oncology forward .
